# Quantifying the impact of treatment delays on breast cancer survival outcomes: a comprehensive meta-analysis

**DOI:** 10.1007/s11357-025-01719-1

**Published:** 2025-06-10

**Authors:** Zoltan Ungvari, Mónika Fekete, Annamaria Buda, Andrea Lehoczki, Gyöngyi Munkácsy, Paola Scaffidi, Tiziana Bonaldi, János Tibor Fekete, Giampaolo Bianchini, Péter Varga, Anna Ungvari, Balázs Győrffy

**Affiliations:** 1 Vascular Cognitive Impairment, Neurodegeneration and Healthy Brain Aging Program, Department of Neurosurgery, University of Oklahoma Health Sciences Center, Oklahoma City, OK USA; 2 Stephenson Cancer Center, University of Oklahoma, Oklahoma City, OK USA; 3 Oklahoma Center for Geroscience and Healthy Brain Aging, University of Oklahoma Health Sciences Center, Oklahoma City, OK USA; 4 Department of Health Promotion Sciences, College of Public Health, University of Oklahoma Health Sciences Center, Oklahoma City, OK USA; 5 International Training Program in Geroscience, Doctoral College, Health Sciences Division/Institute of Preventive Medicine and Public Health, Semmelweis University, Budapest, Hungary; 6 Institute of Preventive Medicine and Public Health, Semmelweis University, Budapest, Hungary; 7 Jozsef Fodor Center for Prevention and Healthy Aging, Semmelweis University, Budapest, Hungary; 8 Doctoral College, Health Sciences Division, Semmelweis University, Budapest, Hungary; 9 Dept. of Bioinformatics, Semmelweis University, 1094 Budapest, Hungary; 10 Cancer Biomarker Research Group, Institute of Molecular Life Sciences, HUN-REN Research Centre for Natural Sciences, 1117 Budapest, Hungary; 11 Department of Experimental Oncology, European Institute of Oncology (IEO) IRCCS, Milan, Italy; 12 Department of Medical Oncology, IRCCS Ospedale San Raffaele, Milan, Italy; 13 Vita-Salute San Raffaele University, Milan, Italy; 14 Dept. of Biophysics, Medical School, University of Pecs, 7624 Pecs, Hungary

**Keywords:** Treatment delay, All-cause mortality, Breast cancer–specific mortality, Survival outcomes, Cancer prognosis, Hazard ratio, Mortality risk

## Abstract

Treatment delay in breast cancer care represents a significant concern in oncology, potentially impacting patient survival outcomes. While various factors can contribute to delayed treatment initiation, the quantitative relationship between specific delay intervals and survival remains incompletely understood in breast cancer management. Our study aims to explore the impact of treatment delays on survival outcomes in breast cancer. A comprehensive literature search was conducted in PubMed, Scopus, and Web of Science databases, covering publications from 2000 to 2025. From an initial 6222 records, 18 eligible studies comprising 25 cohorts were included. Hazard ratios (HRs) for all-cause and breast cancer–specific mortality were extracted or calculated for treatment delays of 4, 8, and 12 weeks. Random-effects meta-analyses were performed, and heterogeneity and publication bias were assessed using *I*^2^ statistics, funnel plots, and Egger’s test. This meta-analysis revealed progressively increasing mortality risks with longer treatment delays. For all-cause mortality, HRs increased from 1.12 (95% CI 1.08–1.15) at 4 weeks to 1.25 (95% CI 1.17–1.33) at 8 weeks, and 1.39 (95% CI 1.26–1.53) at 12 weeks. Breast cancer–specific mortality showed more pronounced effects, with HRs of 1.20 (95% CI 1.06–1.36), 1.43 (95% CI 1.11–1.84), and 1.71 (95% CI 1.18–2.49) for 4-, 8-, and 12-week delays, respectively. Analyses combining both survival outcomes demonstrated consistent risk elevation across all time intervals (4 weeks: HR = 1.12, 95% CI 1.09–1.16; 8 weeks: HR = 1.26, 95% CI 1.18–1.34; 12 weeks: HR = 1.41, 95% CI 1.29–1.55). While heterogeneity was significant (*I*^2^ = 54–92%), no substantial publication bias was detected. Delays in initiating breast cancer treatment are associated with significantly worse survival, particularly for cancer-specific mortality. Each additional 4-week delay increases the hazard of death by over 10%, underscoring the urgency of minimizing delays in diagnosis-to-treatment pathways. These findings have critical implications for healthcare systems, clinical decision-making, and public health policy.

## Introduction

Timely initiation of cancer treatment is a cornerstone of effective oncologic care [1], particularly for high-prevalence cancers such as breast cancer. Despite advances in screening and therapeutics, breast cancer remains one of the leading causes of cancer-related death worldwide [2–4]. A growing body of evidence suggests that even modest delays between diagnosis and treatment can significantly impact survival outcomes [1, 5, 6]. However, treatment delays remain common in clinical practice, driven by a combination of patient-, provider-, and system-level barriers [7–31].

Breast cancer treatment delays can occur at multiple stages of care—from delayed biopsy or imaging, to prolonged scheduling for surgery, chemotherapy, or radiation [6, 23, 26, 32]. Patient-level contributors include low health literacy and low symptom awareness, fear of diagnosis or treatment, limited social support, logistical barriers, and disparities in access to care [6, 23, 26, 32]. Psychological factors, including denial and anxiety [33, 34], can lead to delayed presentation, while logistical issues such as transportation difficulties, work obligations, and caregiving responsibilities disproportionately affect underserved populations [11, 14, 21, 35–38]. These barriers are magnified among older adults, who may also face age-related vulnerabilities such as frailty, multimorbidity, and cognitive decline—all of which complicate treatment planning and contribute to longer decision-making intervals [39]. At the healthcare system level, delays are often due to fragmented care pathways and capacity constraints [6, 23, 26, 32]. Long wait times for specialist consultations, imaging, biopsy, and operating room availability can create bottlenecks. Even within high-resource settings, inefficiencies in referral systems and lack of care coordination can cause treatment to be initiated later than clinically optimal [40, 41]. Importantly, the COVID-19 pandemic has magnified these delays on a global scale, creating unprecedented backlogs in elective surgeries and oncologic services, including disruptions in chemotherapy and radiation therapy scheduling [8, 42–45].

Although prior studies have reported that delayed treatment initiation is associated with increased mortality in breast cancer [46–48], the magnitude and consistency of this effect across different delay durations and survival outcomes remain unclear. Furthermore, while many studies focus on a single treatment modality or endpoint, few provide a comprehensive quantitative synthesis of delay-associated risks across multiple time intervals and mortality outcomes.

Given the substantial burden of breast cancer and the modifiable nature of treatment delays, there is an urgent need to quantify the survival impact of these delays to guide clinical and policy interventions. To address this gap, we conducted a meta-analysis to evaluate how treatment delays of 4, 8, and 12 weeks affect both all-cause and breast cancer–specific mortality. Our findings aim to inform evidence-based benchmarks for acceptable time-to-treatment and support efforts to streamline cancer care delivery systems worldwide.

## Methods

### Data collection and study selection

To ensure a comprehensive evaluation, we conducted a systematic search of the PubMed, Scopus, and Web of Science databases, considering publications from 2000 to 2025. The search strategy included the keywords “breast cancer” AND “treatment delay” AND “mortality OR survival.” In addition, we sourced studies from earlier meta-analyses to complement our research [46–48].

Studies were selected based on predefined inclusion and exclusion criteria. We included prospective cohort studies and retrospective studies that analyzed the effect of treatment delays on breast cancer mortality [7, 11, 49–64]. Studies were required to provide hazard ratios (HRs) or odds ratios (ORs) quantifying the relationship between treatment delays and overall survival outcomes. Additionally, all included studies had to report a follow-up period of at least 30 days to ensure sufficient observation time for meaningful survival analysis.

Conversely, we excluded animal studies, in vitro research, and theoretical models, as these do not directly inform clinical practice in human populations. Studies published in languages other than English were also excluded to maintain consistency in data interpretation. Furthermore, studies with insufficient validity or inappropriate patient populations were excluded to ensure the robustness of our findings.

### Data extraction and quality assessment

Data extraction was performed systematically by two independent researchers to minimize bias and enhance reliability. For each study, key data points were collected, including study characteristics (author names, publication year, and country), cancer type and treatment modality (surgery, chemotherapy, or radiotherapy), hazard ratios (HRs) reflecting the link between treatment delays and survival outcomes, and the degree of treatment delay reported. This information enabled comprehensive comparisons across studies and highlighted the impact of timely treatment on patient outcomes. In cases where discrepancies arose during data extraction, consensus was achieved through discussion between the two researchers. This collaborative approach ensured that all data points were accurately and consistently recorded, thereby enhancing the quality and reliability of the meta-analysis.

### Statistical analyses

We applied two distinct methods to calculate the hazard ratio (HR) as the primary outcome variable in our meta-analysis of breast cancer studies. In cases where a reference period was not provided, the reported HR or OR values were standardized using the formula: HR per *X*-month delay = (HR per 4-week delay)^(*X*-week delay/4-week delay) [6]. For studies that included a defined reference time, we employed a weighted linear regression to evaluate the relationship between treatment delay (in weeks) and the log-transformed hazard ratio (HR) for patient outcomes. Hazard ratio estimates with corresponding 95% confidence intervals (CIs) were calculated for delays of 4, 8, and 12 weeks.

To estimate aggregated risk measures, particularly hazard ratios and their associated 95% CIs, we utilized a random-effects model. This method accounts for variability across studies, thereby enhancing the generalizability of our findings. Forest plots were generated to visually display individual study results alongside the overall summary estimate, facilitating data interpretation and aiding in the identification of potential heterogeneity between studies. All statistical analyses were conducted using the online platform MetaAnalysisOnline.com [65].

### Evaluation of variability and publication bias

To assess inter-study variability, we used Cochran’s *Q* test and the *I*^2^ statistic. Cochran’s *Q* test, based on a chi-squared distribution, was employed to determine whether observed differences in effect sizes exceeded those expected by chance. The *I*^2^ statistic quantified the proportion of total variance attributable to actual study differences rather than random fluctuations.

To investigate potential publication bias, we constructed funnel plots to graphically represent the relationship between study effect sizes and their precision. Asymmetry in these plots may suggest the presence of bias. Additionally, Egger’s regression analysis was performed to statistically evaluate the correlation between effect sizes and their standard errors, providing a quantitative measure of publication bias.

### Subgroup analyses

Additional analyses were conducted to explore potential variations in effect estimates across different endpoints, including overall survival and breast cancer–specific survival. For each subgroup, pooled effect estimates and heterogeneity metrics were calculated to evaluate the specific impact within each category. Furthermore, we extended our analyses to the combined cohort, enabling a comprehensive assessment of overall effects across all included cases. These analyses aimed to provide deeper insights into how treatment delays might differentially affect various patient outcomes in breast cancer.

## Results

### Study selection

**Fig. 1:**
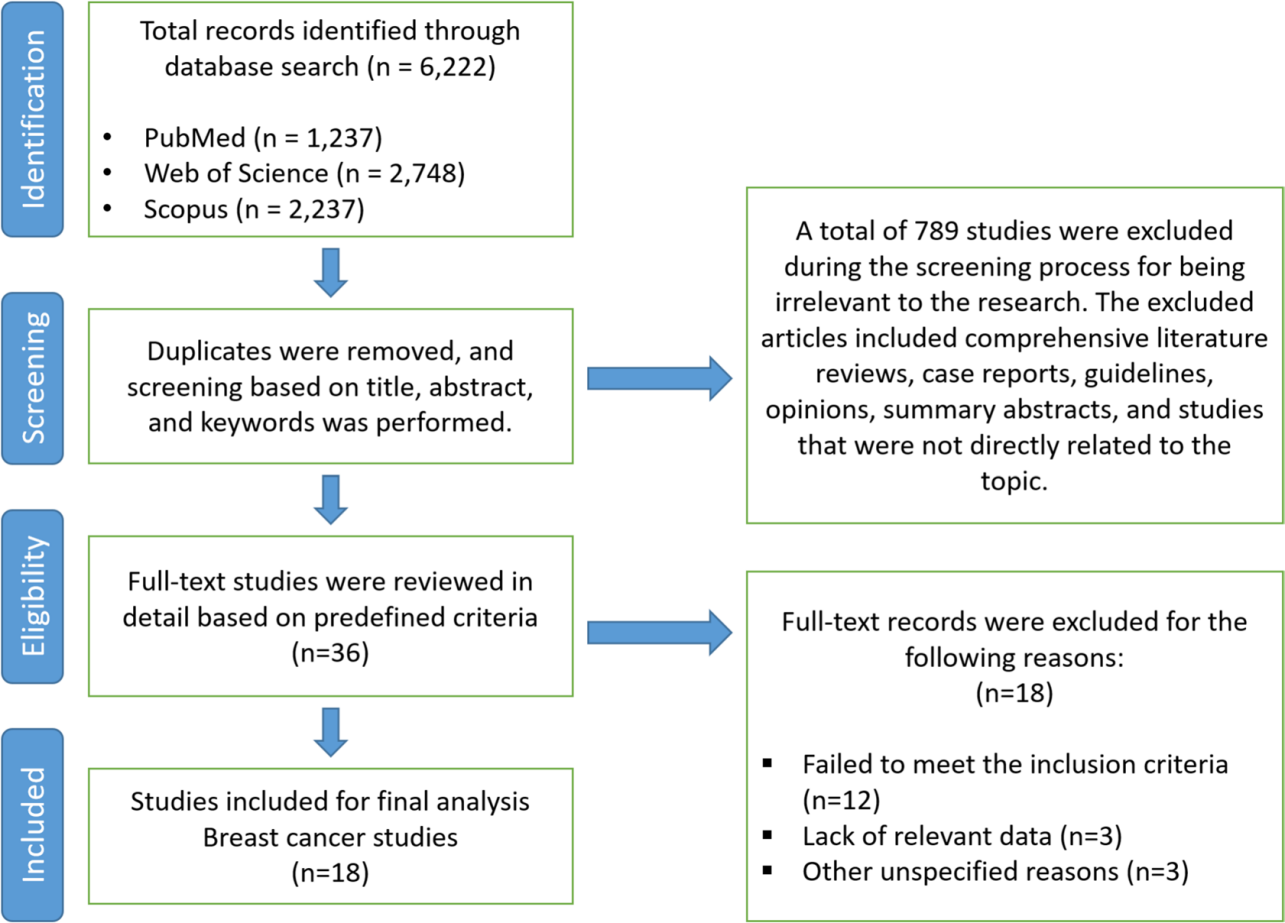
Flow diagram depicting the study selection process

A systematic literature search was conducted across three electronic databases (PubMed, Web of Science, and Scopus), yielding 6222 potentially relevant records (Fig. 1). A total of 789 studies were excluded during the screening process, comprising comprehensive literature reviews, case reports, guidelines, opinions, summary abstracts, and studies not directly related to the research topic. After removal of duplicates and initial title-based screening, 36 articles remained. Of these, 18 articles were excluded due to failure to meet inclusion criteria (*n* = 12), lack of relevant data (*n* = 3), and other reasons (*n* = 3). The remaining 18 studies focusing on breast cancer were included in the final analysis.

Our analysis encompassed a consistent dataset across all delay intervals: 20 cohorts examining all-cause mortality, five cohorts investigating breast cancer–specific mortality, and 25 cohorts for the combined survival analysis. A comprehensive summary of studies investigating treatment delay impacts on survival outcomes is presented in Table 1.

**Table 1:** A summary of studies evaluating the effect of treatment delay on survival outcomes in breast cancer patients. Abbreviations: *CI*, confidence interval; *HR*, hazard ratio; *OR*, odds ratio

Authors	Year	Mortality	4 weeks-delay			8 weeks-delay			12 weeks-delay			HR/OR	Study *N*
			**Rate**	**95% CI**		**Rate**	**95% CI**		**Rate**	**95% CI**			
Bleicher et al	2016	All-cause	1.04	1.03	1.06	1.09	1.06	1.13	1.14	1.09	1.20	HR	94 544
Bleicher et al	2016	Cancer-specific	1.11	1.01	1.22	1.24	1.02	1.50	1.38	1.03	1.83	HR	94 544
Eaglehouse et al	2019	All-cause	1.18	0.98	1.43	1.40	0.96	2.05	1.66	0.94	2.94	HR	9 669
Flores-Balcázar et al	2020	All-cause	1.44	1.23	1.81	2.08	1.52	3.28	3.00	1.87	5.95	HR	720
Freeman et al	2024	All-cause	1.10	1.09	1.11	1.21	1.19	1.24	1.34	1.29	1.38	OR	531 644
Gagliato et al	2014	All-cause	1.22	1.06	1.42	1.50	1.12	2.02	1.84	1.18	2.87	HR	6 827
Hébert-Croteau et al	2004	All-cause	0.96	0.94	0.99	0.93	0.88	0.97	0.89	0.83	0.96	HR	1 062
Hershman et al	2006	All-cause	1.07	0.98	1.16	1.14	0.96	1.35	1.21	0.94	1.56	HR	5 003
Hershman et al	2006	Cancer-specific	1.04	0.87	1.24	1.08	0.76	1.53	1.12	0.66	1.89	HR	5 003
Kupstas et al	2019	All-cause	1.06	1.05	1.08	1.13	1.10	1.16	1.20	1.15	1.25	OR	172 043
Mateo et al	2020	All-cause	1.10	1.08	1.13	1.21	1.17	1.28	1.33	1.26	1.44	HR	351 087
Pathak et al	2023	All-cause	1.19	1.11	1.28	1.41	1.22	1.63	1.68	1.35	2.08	HR	31 306
Polverini et al	2016	All-cause	0.99	0.96	1.03	0.99	0.93	1.05	0.98	0.90	1.08	HR	420 792
Sanford et al	2016	All-cause	1.00	0.80	1.27	1.01	0.63	1.60	1.01	0.50	2.03	HR	1 101
Shih et al	2022	All-cause	0.90	0.66	1.21	0.80	0.44	1.46	0.72	0.29	1.76	HR	49 426
Shin et al	2013	All-cause	1.21	1.04	1.42	1.47	1.08	2.01	1.79	1.13	2.84	HR	2 045
Smith et al	2013	All-cause	1.27	1.02	1.58	1.60	1.03	2.49	2.03	1.05	3.93	HR	8,860
Trufelli et al	2015	All-cause	1.19	1.14	1.24	1.42	1.31	1.54	1.69	1.50	1.91	HR	348
Yun et al	2012	All-cause	1.59	1.37	1.84	2.53	1.88	3.39	4.02	2.57	6.23	HR	147 682
Yung et al. (all treatment)	2020	All-cause	1.52	1.22	1.87	2.30	1.50	3.50	3.49	1.84	6.55	HR	3 368
Yung et al. (all treatment)	2020	Cancer-specific	1.73	1.23	2.47	3.00	1.51	6.12	5.20	1.86	15.14	HR	3 368
Yung et al. (chemotherapy)	2020	All-cause	1.18	1.01	1.38	1.39	1.02	1.90	1.64	1.03	2.62	HR	3 368
Yung et al. (chemotherapy)	2020	Cancer-specific	1.31	1.03	1.66	1.71	1.07	2.75	2.24	1.11	4.56	HR	3 368
Yung et al. (radiotherapy)	2020	All-cause	1.09	0.99	1.19	1.19	0.99	1.42	1.30	0.99	1.69	HR	3 368
Yung et al. (radiotherapy)	2020	Cancer-specific	1.22	1.00	1.49	1.49	1.00	2.21	1.82	1.00	3.29	HR	3 368

### Effect of 4-week delay in treatment

**Fig. 2:**
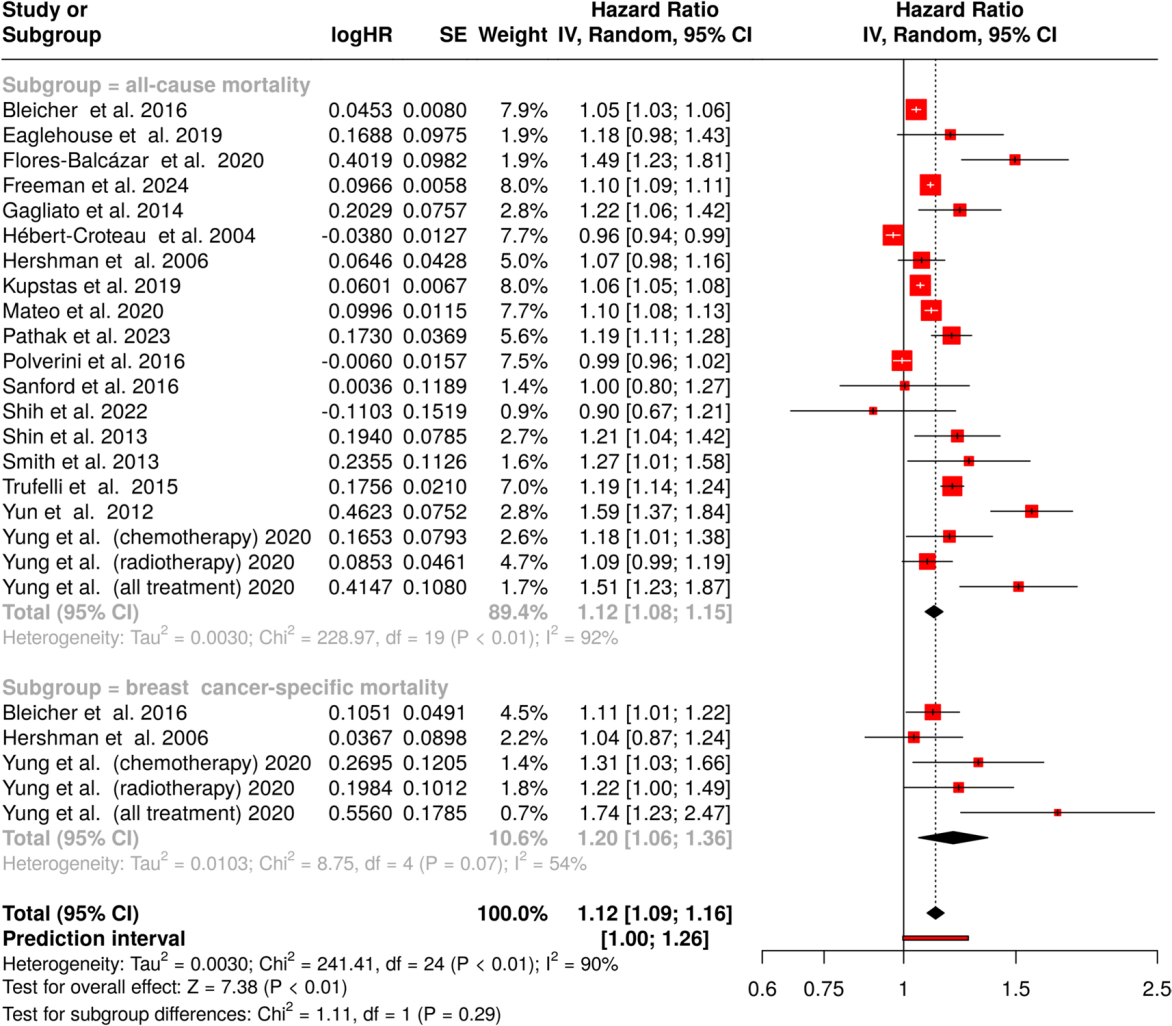
Impact of a 4-week treatment delay on mortality in breast cancer patients. The upper panel depicts all-cause mortality, while the lower panel presents breast cancer–specific mortality. Hazard ratios (HRs) and 95% confidence intervals (CIs) were estimated using a random-effects model. Each square represents an individual study estimate, with the size proportional to its statistical weight, while horizontal lines indicate the corresponding CIs. The black diamond represents the pooled effect estimate. Heterogeneity across studies is quantified using the *I*^2^ statistic for both subgroups and for all studies. Abbreviations: CI, confidence interval; HR, hazard ratio; IV, inverse variance; SE, standard error

Investigation of all-cause mortality using a random effects model with inverse variance method revealed a statistically significant increase in mortality risk (HR = 1.12, 95% CI 1.08–1.15) as depicted in Fig. 2, upper panel. Significant heterogeneity was detected among studies (*p* < 0.01), with a high *I*^2^-value (*I*^2^ = 92%), indicating that most variability arose from true heterogeneity rather than random chance.

Analysis of breast cancer–specific mortality demonstrated a more pronounced effect on survival outcomes (HR = 1.20, 95% CI 1.06–1.36). Moderate heterogeneity was observed (*I*^2^ = 54%, *p* = 0.07), suggesting varying effects across studies (Fig. 2, lower panel). The comprehensive evaluation combining both survival outcomes across all 25 studies demonstrated a significant impact on survival (HR = 1.12, 95% CI 1.09–1.16), with substantial heterogeneity (*I*^2^ = 90%, *p* < 0.01).

### Effect of 8-week delay in treatment

**Fig. 3:**
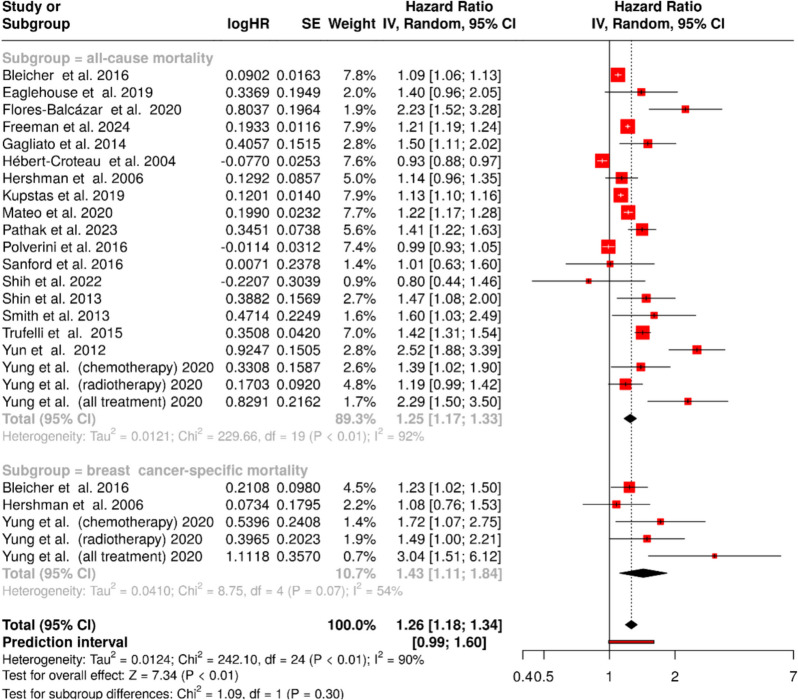
Association between an 8-week treatment delay and mortality in breast cancer patients. Abbreviations: CI, confidence interval; HR, hazard ratio; IV, inverse variance; SE, standard error

Extended treatment delay of 8 weeks demonstrated a more substantial impact on all-cause mortality (HR = 1.25, 95% CI 1.17–1.33). Statistical assessment revealed significant heterogeneity (*p* < 0.01), with an *I*^2^-value of 92%, indicating considerable variation in effect sizes across studies (Fig. 3, upper panel). The analysis of breast cancer–specific mortality showed an even more pronounced effect (HR = 1.43, 95% CI 1.11–1.84), though heterogeneity remained moderate (*I*^2^ = 54%, *p* = 0.07) (Fig. 3, lower panel).

The combined analysis of both survival outcomes revealed a significant elevation in mortality risk (HR = 1.26, 95% CI 1.18–1.34). Heterogeneity remained substantial across the combined dataset (*I*^2^ = 90%, *p* < 0.01), suggesting consistent variation in treatment effects across different study populations and settings.

### Effect of 12-week delay in treatment

**Fig. 4:**
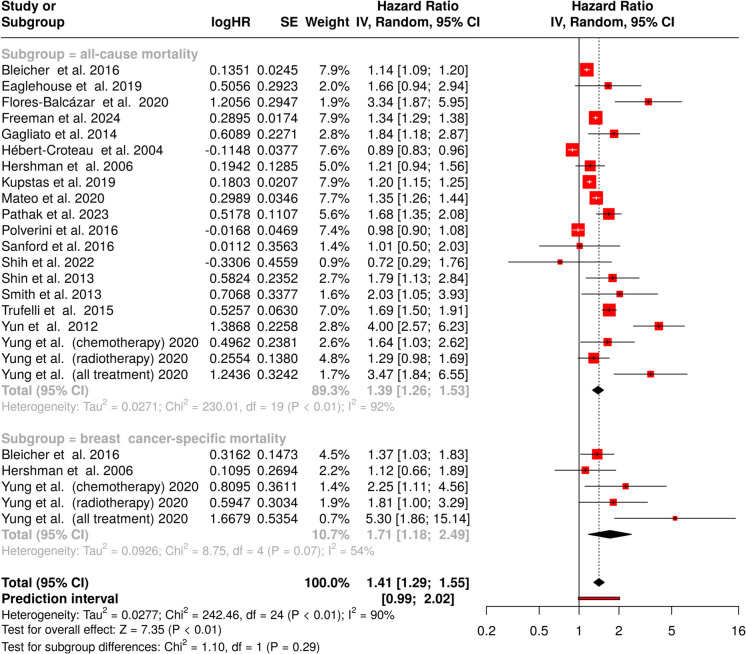
Effect of a 12-week treatment delay on mortality in breast cancer patients. The upper panel shows all-cause mortality, with a highly significant pooled hazard ratio of 1.39 (95% CI 1.26–1.53). The lower panel presents breast cancer–specific mortality, with a pooled HR of 1.71 (95% CI 1.18–2.49). Abbreviations: CI, confidence interval; HR, hazard ratio; IV, inverse variance; SE, standard error

The lengthiest delay period demonstrated the most pronounced impact on survival outcomes. All-cause mortality analysis revealed a substantial increase in risk (HR = 1.39, 95% CI 1.26–1.53), maintaining significant heterogeneity (*I*^2^ = 92%, *p* < 0.01) as shown in the upper panel of Fig. 4. The evaluation of breast cancer–specific mortality showed the highest risk elevation among all analyzed time points (HR = 1.71, 95% CI 1.18–2.49), with moderate heterogeneity persisting (*I*^2^ = 54%, *p* = 0.07) (Fig. 4, lower panel).

The comprehensive analysis combining both survival measures demonstrated a marked increase in overall mortality risk (HR = 1.41, 95% CI 1.29–1.55). Significant heterogeneity remained evident (*I*^2^ = 90%, *p* < 0.01), reflecting consistent variation in effect magnitude across the included studies.

### Publication bias

**Fig. 5:**
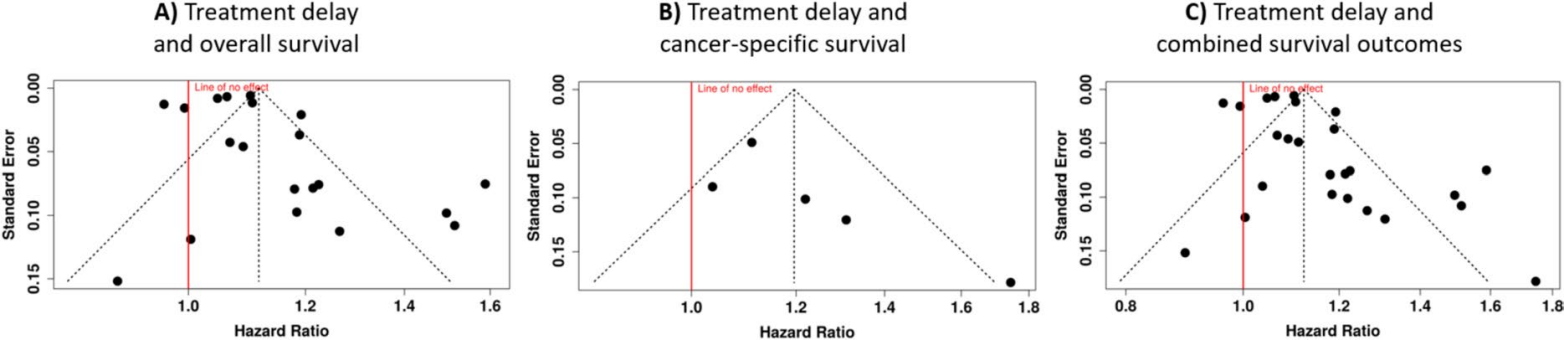
Funnel plots assessing publication bias in the meta-analysis of the effect of treatment delay on mortality in breast cancer patients. **A** Analysis of all-cause mortality across 20 studies; **B** analysis of breast cancer–specific mortality in five studies; **C** combined analysis of both survival outcomes across all 25 studies. The *x*-axis represents hazard ratios, while the *y*-axis shows the standard error. The vertical red line indicates no effect (HR = 1.0), and the dotted lines represent the 95% confidence intervals

A publication bias occurs when studies with statistically significant or positive results are more likely to be published than those with non-significant or negative findings. This can lead to an overestimation of the effect size in a meta-analysis, as smaller studies with less precise estimates might be missing or underrepresented. We conducted a thorough evaluation of potential publication bias using both visual and statistical approaches. Funnel plots were employed to examine the relationship between study effect sizes and their standard errors, with asymmetry potentially indicating selective reporting or small-study effects (Fig. 5). Visual examination of funnel plots was supplemented with Egger’s test for statistical verification of asymmetry.

For all-cause mortality analysis (20 cohorts), the funnel plot showed no indication of publication bias (Fig. 5A), supported by Egger’s test results (intercept 1.34, 95% CI − 0.67–3.35, *t* = 1.304, *p* = 0.209). Similarly, examination of breast cancer–specific mortality (five cohorts) revealed no significant publication bias (Fig. 5B), confirmed by Egger’s test findings (intercept 2.46, 95% CI 0.13–4.79, *t* = 2.067, *p* = 0.131).

The combined analysis of both survival outcomes with all 25 cohorts maintained symmetrical distribution in funnel plot assessment (Fig. 5C), with Egger’s test results supporting the absence of significant publication bias (intercept 1.35, 95% CI − 0.19–2.90, *t* = 1.714, *p* = 0.100).

## Discussion

This systematic review and meta-analysis demonstrate a consistent and clinically significant association between delays in breast cancer treatment initiation and increased mortality. We observed that each 4-week increment in treatment delay led to a progressively higher risk of death, with the effect being more pronounced for breast cancer–specific mortality compared to all-cause mortality. Notably, a 12-week delay was associated with a 41% increased risk of death from any cause and a 71% increase in breast cancer–specific mortality, underscoring the importance of timely treatment across all stages of care.

These findings build on previous research by providing a robust, quantitative synthesis of delay-related risk across multiple time intervals. Earlier studies have reported mixed results depending on cancer subtype and healthcare settings [7, 11, 46–64]. Our analysis confirms that even modest delays significantly compromise survival and supports the assertion that time-to-treatment is a modifiable factor that directly affects patient outcomes in breast cancer.

The more pronounced effect on cancer-specific mortality suggests that treatment delays primarily affect disease progression and tumor-related outcomes, rather than comorbid conditions. This is particularly relevant in the context of hormone receptor-negative or aggressive subtypes such as triple-negative and HER2-positive breast cancer [66–68], where tumor doubling time is shorter and early intervention is crucial. Although some early-stage, hormone receptor–positive tumors may be more biologically indolent, our findings indicate that even these patients are not exempt from increased risk with treatment deferral. These observations support the need for individualized triage strategies based on both tumor biology and patient vulnerability—prioritizing high-risk subtypes while avoiding unnecessary delays in lower-risk cases. While our meta-analysis included diverse treatment modalities and patient populations, the consistent dose–response relationship across delay intervals strengthens the biological plausibility that delays facilitate tumor progression, micrometastasis, and diminished treatment efficacy. The heterogeneity observed across studies, particularly in all-cause mortality, likely reflects variation in study design, treatment types, healthcare systems, and patient characteristics. Nonetheless, the direction and magnitude of effect remained consistent, reinforcing the conclusion that treatment delay is detrimental to survival.

These findings highlight the urgent need to address the multifactorial causes of treatment delay in breast cancer, which span patient-level factors (e.g., socioeconomic status, health literacy, psychological barriers, social support [69]), provider-level issues (e.g., referral patterns, diagnostic decision-making, communication gaps), and system-level challenges (e.g., workforce shortages, geographic disparities, and fragmentation of care) [27, 29]. Given the clear association between treatment delays and increased mortality, coordinated efforts across all levels of care are essential. At the system level, interventions such as streamlined referral pathways, centralized scheduling, and fast-track diagnostic and treatment programs have shown promise in reducing bottlenecks, particularly in surgical and oncologic services [29]. Integrated breast cancer care pathways, multidisciplinary coordination, and the use of trained patient navigators can further enhance care continuity and reduce administrative barriers [70–72]. Expanding telemedicine for pre-treatment consultations may also improve timely access to care, especially in rural or underserved populations [73–75]. At the provider level, raising awareness about the clinical impact of delays and implementing standardized time-to-treatment benchmarks can foster a sense of urgency and accountability [76–80]. For older adults and medically complex patients, incorporating geriatric assessment tools and prehabilitation programs can facilitate timely treatment initiation while ensuring personalized care [81–87]. Incorporating these strategies into national cancer control plans and institutional quality metrics may help build a more responsive, equitable, and efficient breast cancer care continuum. Our study also provides timely evidence for policymakers and healthcare systems, particularly in light of widespread delays caused by the COVID-19 pandemic [88–90]. Disruptions to surgical scheduling, diagnostic services, and oncologic follow-up have resulted in significant backlogs in cancer care [90]. The quantitative risk estimates provided here may help guide prioritization strategies, triage protocols, and resource allocation in times of healthcare strain.

Limitations of this analysis include the reliance on observational data, which may be subject to residual confounding despite multivariable adjustments in most included studies. While many cohorts adjusted for key prognostic factors such as stage, age, and comorbidities, unmeasured confounders—including socioeconomic status, healthcare access, or patient preferences—may still influence the observed associations. Additionally, there was heterogeneity in the definition and measurement of treatment delay across studies, and endpoints were not always reported uniformly. The smaller number of cohorts reporting breast cancer–specific mortality limited our ability to explore this outcome with the same precision as all-cause mortality, although the observed effect was consistently larger in this subgroup. We also did not conduct in-depth stratified analyses by treatment modality (e.g., surgery vs. chemotherapy) or by molecular subtype (e.g., triple-negative, HER2-positive, or HR-positive disease), which could refine risk estimates for different clinical scenarios. Our analysis included studies evaluating delays in surgery, chemotherapy, and radiation therapy. However, treatment modality–specific data were not consistently disaggregated, and therefore, we were unable to perform a stratified meta-analysis by treatment type. The effect of delay may well differ across modalities—for example, primary surgery versus adjuvant systemic therapy—and future meta-analyses should explore these distinctions where data permit. Furthermore, our analysis does not distinguish between intentional and unintentional delays—for example, those related to patient choice versus system-level inefficiencies. This lack of granularity limited our ability to assess whether the source of delay influenced outcomes. It is plausible that these categories exert differential effects, particularly in settings where delays are caused by infrastructure limitations rather than clinical indecision or patient-related factors. Future research would benefit from standardized reporting of delay origin to enable stratified analyses. Finally, while our study included diverse populations from multiple countries, generalizability may still be limited in lower-resource settings where delays are more common and treatment access is variable.

In conclusion, our findings emphasize that even short delays in breast cancer treatment initiation have significant consequences for survival. The data presented here support the prioritization of timely cancer care and may inform clinical benchmarks and health system performance indicators. Ensuring rapid and equitable access to breast cancer treatment should remain a public health priority globally.
